# Too many wild boar? Modelling fertility control and culling to reduce wild boar numbers in isolated populations

**DOI:** 10.1371/journal.pone.0238429

**Published:** 2020-09-18

**Authors:** Simon Croft, Barbara Franzetti, Robin Gill, Giovanna Massei

**Affiliations:** 1 National Wildlife Management Centre, Animal and Plant Health Agency, York, United Kingdom; 2 Institute for Environmental Protection and Research (ISPRA), Rome, Italy; 3 Centre for Ecosystems, Society and Biosecurity, Forest Research, Farnham, United Kingdom; Universitat Autonoma de Barcelona, SPAIN

## Abstract

Wild boar and feral swine number and range are increasing worldwide in parallel with their impact on biodiversity and human activities. The ecological and economic impact of this species include spread of diseases, vehicle collisions, damage to crops, amenities and infrastructures and reduction in plant and animal abundance and richness. As traditional methods such as culling have not contained the growth and spread of wild boar and feral pigs, alternative methods such as fertility control are now advocated. We used empirical data on two isolated wild boar populations to model and compare the effects of different regimes of culling and fertility control on population trends. We built a Bayesian population model and applied it to explore the implications for population control of various management options combining culling and/or contraception. The results showed that, whilst fertility control on its own was not sufficient to achieve the target reduction in wild boar number, adding fertility control to culling was more effective than culling alone. In particular, using contraceptives on 40% of the population to complement the culling of 60% of the animals, halved the time to achieve our target reduction compared with culling only. We conclude that, assuming the effort of adding fertility control to culling was found to be cost-effective in terms of population reduction, these two methods should be used simultaneously if a rapid decrease in wild boar number is required for a closed population.

## Introduction

Wild boar and feral swine belong to the same species *Sus scrofa* and are among the most widely distributed large mammals in the world, where they occur as native or introduced [1–3]. In the last decades, the number and range of these animals have increased dramatically, due to the adaptability of the species to a variety of habitats, but also due to mild winters, reforestation, increased availability of crops, supplementary feeding or baiting and introductions by hunters [4–6]. In particular, wild boar and feral swine (hereafter referred with either term, in relation to specific studies) have the highest reproductive rate among ungulates, with annual population growth rates that may exceed 2.0 [7, 8].

The environmental and economic impacts of this species include spread of diseases to livestock and people, vehicle collisions, damage to crops, amenities and infrastructures and reduction in plant and animal abundance and richness (e.g. [9–14]). Wild boar have also colonised urban areas, where their impact include extensive damage to private gardens, public parks, sport grounds and cemeteries, as well as transmission of diseases to humans and companion animals (e.g. [15, 16]). Reducing local densities is generally assumed to decrease the species’ impacts (e.g. [17]) and in recent years, outbreaks of diseases such as African swine fever (ASF) and classical swine fever (CSF), which may cost affected countries billions of euros (e.g. [18, 19]), have catalysed discussions on options to reduce local wild boar numbers.

Best-practice guidelines and eradication programmes recommend that several methods should be included to manage wild boar and feral swine (e.g. [20–22]). Shooting, trapping and toxicants have traditionally been employed to control wild boar and feral swine numbers [8, 20, 23]. However, traditional hunting is declining in most countries and the recent increase in densities and range of wild boar indicate that hunters are unable to contain this species [6, 24, 25]. To discourage illegal introductions, some areas also preclude the hunting and trapping of wild boar and feral swine except as authorized by appropriate officials [26, 27]. Toxicants can be used as a cost-effective means of reducing pig populations rapidly over large areas (e.g. [28, 29]) and new compounds, specifically developed for feral swine, are now commercially available (e.g. [30]). However, in most countries there are no registered toxicants to control this species and the use of these compounds is often opposed on grounds of animal welfare and environmental impact. In some contexts, such as urban areas and national parks, lethal methods to regulate wild boar numbers can be unpopular, logistically unfeasible or even illegal [31]. In recent years, fertility control has emerged as a non-lethal alternative or a complementary option to culling especially where there is little public support for hunting or lethal control [32–34].

We examined the effects of culling and fertility control on wild boar numbers, as these methods are most likely to be considered for reducing wild boar numbers in contexts where toxicants are not registered or allowed. Whilst several studies modelled the effects of culling on wild boar population dynamics (e.g. [7, 8, 25, 35, 36]), few [37, 38] were based on empirical data on actual numbers of wild boar. In addition, little attention has been paid to evaluating the impact of different levels of both fertility control and culling on wild boar number. A notable exception (Pepin et al. [39]) used empirical data on culling and demographic parameters to model the combined effects of fertility control and culling to manage wild pig populations, although the actual densities of pigs in the areas used for this study was unknown.

We used the data on two similar size, closed populations of wild boar, one in the UK and one in Italy, to estimate the effects of culling and fertility control on wild boar numbers. In particular, we used the long-term dataset on the number of wild boar estimated and removed per year from the Italian population to build the model that was then employed to predict the effects of different population management methods on the size of both populations. The specific objectives were: (i) to develop a model on wild boar population dynamics for a closed population; (ii) to validate this model against empirical data; (iii) to estimate and compare the effects of different regimes of culling and fertility control on wild boar population dynamics; (iv) to specify the effort (in terms of target population reduction percentages) and time required to progressively control the abundance of wild boar by using the two methods separately and in combination.

## Materials and methods

The authority for data collection within the Castelporziano Preserve (Italy) and the Forest of Dean Estate (England) rests with the Institute for Environmental Protection and Research (ISPRA) and the Forestry Commission (FC) respectively. All data for this study was provided by these organisations and they were active co-operators in this research. No animals were used for the specific purposes of this research. All data was collected as a matter of routine population control operations and analysed retrospectively for this study.

### Study area

We obtained data on the number of wild boar from two study areas, the Castelporziano Preserve in Italy and the Forest of Dean in England. The Preserve of Castelporziano (59.9 km^2^) is a fenced protected area along the Tyrrhenian Sea, near Rome in Italy (41°44′N, 12°24′E). The area is almost flat (elevation ranges from -1 to 78 m a.s.l.) and mainly south oriented. It’s covered by mixed deciduous and evergreen woods mainly composed by oak tree (*Quercus* spp.) species, Mediterranean evergreen shrubs, pine woods, xeric vegetation of pastures, wetlands, and dunes. The climate is typically Mediterranean, with mean (±sd) annual rainfall of 731.9 ± 218.3 mm and mean monthly temperatures ranging from 10.6 ± 2.8°C in January to 25.0 ± 2.1°C in August. No sport hunting is allowed but wild boar are harvested every year by the staff of the preserve through summer captures and autumn-winter culling [40].

The Forest of Dean (76.9 km^2^) in Gloucestershire, UK (51°48.4′N, 2°33.1′W) is managed by the Forestry Commission. Broadleaved trees cover approximately 45% of the forest and comprise mainly pedunculate oak *Quercus robur*, with beech *Fagus sylvatica*, sweet chestnut *Castanea sativa*, rowan *Sorbus aucuparia*, holly *Ilex aquifolium*, and sessile oak *Quercus petraea*. The remainder consists of stands which are mixtures of conifers principally larch *Larix decidua*, Norway spruce *Picea abies*, Scots pine *Pinus sylvestris*, Corsican pine *P*. *nigra*, and Douglas fir *Pseudotsuga menziesii*. The climate is temperate with annual rainfall of 745 mm and mean daily temperatures ranging from 5°C in January to 18°C in July. The feral boar population originated from escapes from a farm in the late 1990s [41], and is now monitored annually in all the main forest compartments (7,690 ha). Culling and monitoring of road casualties is carried out by Forestry Commission staff.

### Data collection

In Castelporziano, wild boar births extend between February and September, with a peak in April-June [40]. Since the dry season limits greatly the availability of food for wild boar, counts at feeding sites have been carried out since 1996 in midsummer (end of July), using Capture Mark Recapture (CMR) to estimate population size [40]. Feeding sites (80–88) are distributed randomly across the area (an average of 1.7 feeding sites/km^2^) and baited daily between the end of June and late September to support the survey and the captures of wild boar (see [40, 42, 43] for details). Each year, about 100 animals were captured in late summer, marked with two numbered and coloured ear tags and released.

Since 2001, autumn surveys of wild boar in the Castelporziano Preserve have also been carried out through nocturnal line transect sampling (NLTS) described in Franzetti et al. [42]. Briefly, this method estimates the probability of detecting animals as a function of distances between animals and transects. Counts are hence adjusted over the sampled area as a function of the estimated detectability [44]. As wild boar are more active at night, especially during the mast season, and since the vegetation cover is dense, thermal imaging is used to increase the probability of detecting wild boar groups along transects surveyed between September and October [45, 46]. Latest figures from 2018 estimate the population of boar in the preserve to be between 2,316 and 2,568 based on CMR and NLTS respectively; a density of between 38.6 and 42.9 wild boar per km^2^. The proportion of wild boar culled per year fluctuated between 15% and 55% of the estimated population however typically it is maintained between 30% and 40%.

NLTS using thermal imaging has also been carried out in the Forest of Dean since 2013. In 2018, a total of 137 km of transect were surveyed yielding an estimate of 1,635 boar with a 95% confidence interval ranging from 1,200 to 2,228; an average density of 21.1 wild boar per km^2^. The proportion of wild boar culled per year fluctuated between 15% and 31% of the estimated population with the average maintained close to 25%. The number of boar killed in traffic accidents and found dead was estimated to be approximately 10% of the population [47].

We recognise that the density of boar at both study sites is large and compared with other estimates found in the literature (e.g. densities of ~2 boar/km^2^ for Northern Europe) appears atypical. However, it should be noted that unlike the figures here most estimates of wild boar density are reported after the hunting season and only reflect numbers of adults (i.e. they exclude piglets and juveniles which are included in our numbers). Furthermore, estimates are typically derived from hunting statistics which are known to strongly underestimate the actual densities of this species; providing explanation as to why reported harvests increase gradually from year to year throughout Europe despite a reduction in hunting effort [6].

### Data preparation

**Fig 1:**
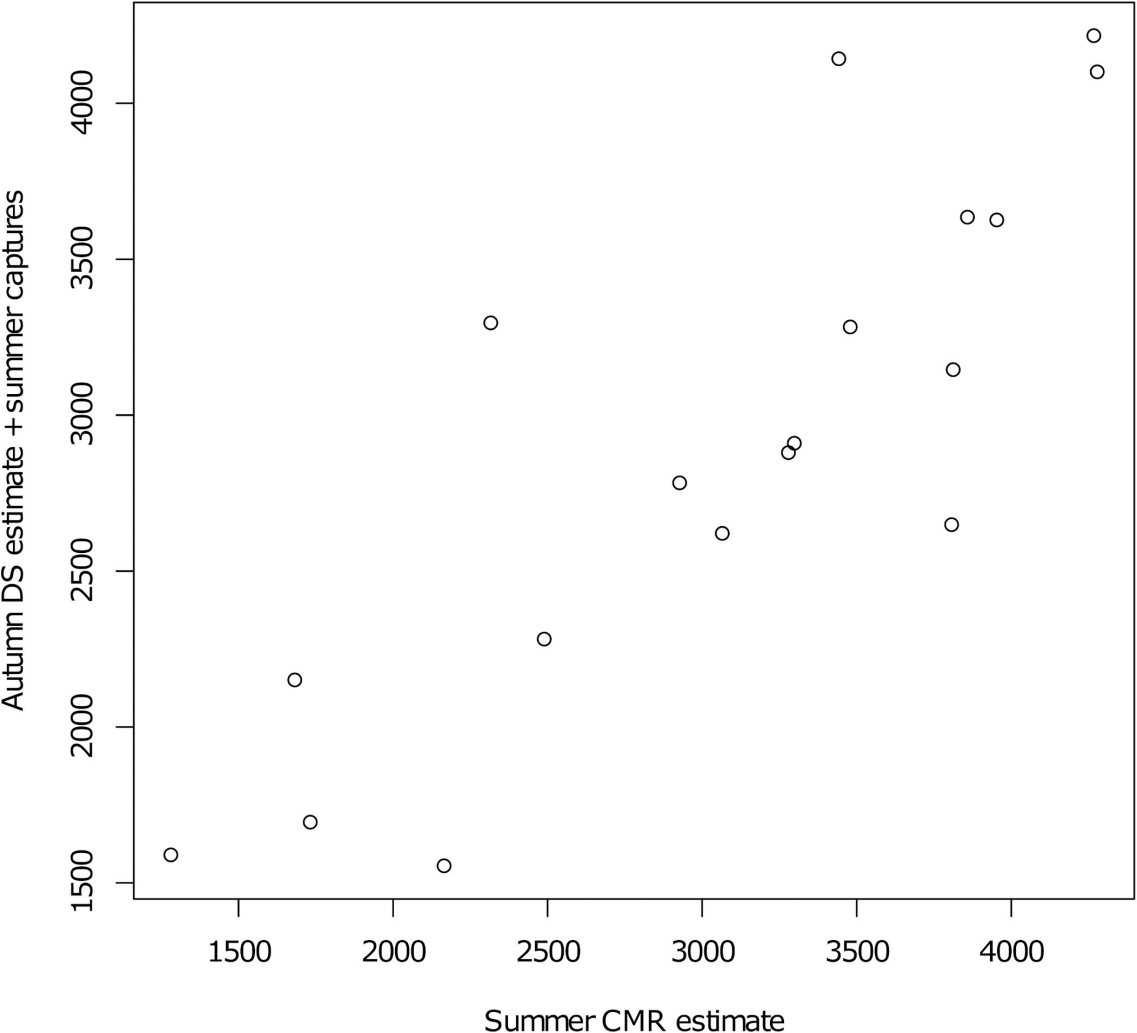
Population estimates in Castelporziano. Comparison of yearly population estimates of wild boar numbers in Castelporziano between 2001 year and 2018 year based on capture-mark-recapture (CMR) and on nocturnal distance sampling (NLTS). CMR was conducted in summer, before animals were trapped and removed. NLTS was conducted in autumn and adjusted to account for number of wild boar trapped and removed in summer.

For the Castelporziano Preserve, we adopted post-breeding, pre-removal (summer live cage trapping and winter shooting) population sizes, i.e. we used the total number of wild boar estimated through NLTS in autumn each year, between 2001 and 2016. This number indicates the density of wild boar post-breeding and post-live cage trapping but pre-cull, adjusted using data from cage trapping in late summer to account for pre-survey removals. We considered these population estimates to be more reliable than those based on data from early summer (post-breeding pre-removal) CMR, which better match the requirement of the model. However, comparison between NLTS and CMR showed good agreement (Fig 1) with Pearson’s correlation coefficient of 0.84 suggesting either could be used for the model.

We derived data on demographic structure from summer counts at feeding stations (post-breeding, pre-removal) describing sex, numbers of juveniles and adults. Focardi et al. [37] established that this method underestimates the number of adult males and through further investigation using CMR analysis that the ratio of adult males to females is approximately 1:1; we thus adjusted the counts of adult males to be equal to that of females. Counts for juveniles and adults were pooled by sex into breeding stage classes. Finally, the proportion of juveniles, females and males were scaled up to population estimates.

We excluded data for 2017 and 2018 from model fitting due to the extreme environmental conditions (long drought) that strongly affected wild boar number and that were not representative of typical conditions observed in the area. These data were instead retained for validation.

### Analysis

Following a similar method to that outlined in Raiho et al. [48] we used a Bayesian model describing the ecology of wild boar in a closed population to obtain posterior distributions of key parameter values. We then applied this derived framework to explore the implications for population control of various management options combining culling and/or contraception.

### Process model

To capture the ecological processes within a closed population of wild boar we developed a stochastic stage-based Lefkovitch matrix model [49] explicitly dividing description into three distinct cohorts: piglets and sexually immature juveniles, all referred to as “juveniles” (<1 year old, as in [35]); sexually mature, adult, females (>1 year old); and, adult males (>1 year old). Deterministically, we defined the model as follows:

$$A_{t}=\left[\begin{array}{ccc} 0 & s_{2}F_{t} & 0\\ s_{1}m & s_{2} & 0\\ s_{1}(1-m) & 0 & s_{3} \end{array}\right]$$ (1)

$$n_{t+1}=A_{t}{\left(n-r\right)}_{t},$$ (2)

where *s*_*i*_, *m*, *F*_*t*_ and ***n-r***, denote the annual survival of boar in each cohort, the sex ratio of juveniles surviving to breeding age, the density-dependant fecundity of females and numbers of boar (post-breeding post-removal) in each cohort respectively. Density-dependant fecundity was defined as follows:

$$F_{t}=f_{0}e^{\frac{-\sum {\left(n-r\right)}_{t}}{k}},$$ (3)

where *f*_*0*_ and *k* represent the maximum number of juveniles per female at zero population and the population carrying capacity (density multiplied by study area) at which the rate of reproduction is half that of the maximum respectively.

To account for any effects not considered in the deterministic model, for example the impact of mast yield, we included a separate stochastic element for each cohort as follows:

$$\log(n_{t+1})∼\mathrm{multivariate}\;\mathrm{normal}\;(\log(A_{t}{\left(n-r\right)}_{t}),\sigma_{i}^{2}),$$ (4)

where *σ_i_* is the process variance for each cohort not represented in our deterministic model.

### Parameterisation and evaluation

We estimated parameter distributions from the literature following an approach similar to that outlined in Holland et al. [50] (Table 1). We fitted parameters describing probabilities (e.g. survival) with lower bound zero and upper bound one to a beta distribution. We fitted parameters describing continuous quantities (e.g. maximum litter size) with lower bound zero but no conceptual upper bound to a gamma distribution. For both, we fitted distributions using the “fitdistr” function from the “MASS” package [51] in R statistical software [52]. In the absence of data to inform estimates, we assigned all remaining parameters to be uniform priors with plausible but broad ranges. For carrying capacity we assumed a lower bound of zero and upper bound of 20,000, equivalent to a densities of about 400 boar/km^2^ (note that carrying capacity is an abstract concept designed to mimic density-dependent behaviour and as such values do not necessarily represent achievable population densities). For each process variance, following Raiho et al. [48], we assumed a lower bound of zero and upper bound of 2.

**Table 1:** Summary of prior parameterisation from existing literature sources.

Parameter	Description	Range (n)	Distribution	References
Fecundity (*f*)	Maximum litter size at low population (based on embryos per sow)	4.3–6.8 (21)	gamma(47.4,8.66)	[7, 53, 54–61]
Sex ratio (*m*)	Proportion of Juveniles reaching adulthood female	0.42–0.62 (11)	beta(31.2,29.2)	[37, 53, 55, 61–63]
Survival female (*s*_*F*_)	Adult (>1yr) female annual survival probability	0.31–0.99 (19)	beta(2.89,1.46)	[7, 37, 57, 64–68]
Survival Juveniles (*s*_*I*_)	Juveniles (<1yr) annual survival probability	0.09–0.99 (21)	beta(1.92,1.54)	[7, 37, 53, 55, 57, 58, 64–68]
Survival male (*s*_*M*_)	Adult (>1yr) male annual survival probability	0.31–0.99 (18)	beta(2.82,1.55)	[7, 37, 57, 64–66, 68]

To estimate posterior parameter distributions of parameters and model predictions we applied a Bayesian fitting approach using Markov chain Monte Carlo (MCMC) methods implemented in JAGS [69] from the R package “rjags” [70]. As in Raiho et al. [48], we chose initial values of chains to be diffuse to the means of the prior distributions [71]. We accumulated 100,000 samples from each of 3 chains following a 10,000 iteration burn-in. We assessed convergence by visual inspection of trace plots (see supplementary; S1 File) and associated Gelman-Rubin diagnostics [72]. Similarly, we tested model fit by visual comparison between observed and predicted data. More formally, we tested for lack of fit defining an appropriate test statistic applied to both observations (*T*^*obs*^) and predictions (*T*^*pred*^) separately which we then combine to calculate a Bayesian P-value (*P*_*B*_) [73] as follows:

$$T^{obs}=\sum {\left(y_{t}-\mu_{t}\right)}^{2}\;T^{pred}=\sum {\left(y_{t}^{pred}-\mu_{t}\right)}^{2},$$ (5)

$$P_{B}=\frac{\sum \left[T^{pred}(y^{pred},\theta )\geq T^{obs}(y,\theta \right)]}{N},$$ (6)

where ***y*** is the set of observed data, ***μ*** is the median model prediction of wild boar populations each year, ***y***^*pred*^ is a single sample model prediction drawn from the posterior distribution based on corresponding parameters *ϴ* and *N* is the number of random samples drawn. As computational cost is low we chose to compute *P*_*B*_ based on the full MCMC; 300,000 samples. Lack of fit is indicated if *P*_*B*_ is close to 0 or 1, i.e. model predictions show consistent bias either under- or over- estimating populations compared to observations suggesting the model framework may contain structural deficiencies.

### Simulation experiments

Using the model framework, we tested various combinations of culling (0, 20, 40, 60 or 80 percent removal from each cohort) and fertility control (0, 20, 40, 60 or 80 percent reduction in females of breeding age) which could be applied to manage wild boar in the Castelporziano Preserve for 20 years, starting after 2018. We conservatively assumed that contraception only lasts for a single year, thus the percentage of animals rendered infertile reflect annual effort, although some contraceptives can induce multi-year infertility after a single dose [33], suggesting that the required effort may in reality be significantly lower. To evaluate the efficacy of control we considered a nominal management objective to reduce and maintain the population at or below 400 individuals, suggested as the critical threshold at which CSF would become self-sustaining within a similar sized (40 km^2^) study area [74]. A similar number of wild boar (500 animals), was also proposed by foresters in Castelporziano to reduce the ecological impact of wild boar.

We repeated the same analysis for the wild boar population in the Forest of Dean, assuming an additional adult mortality of 10% per year due to road traffic accidents (RTAs) not present in Castelporziano. Initial population sizes were scaled to match reported estimates [47]. Otherwise, the two study sites were considered to be identical in terms of area covered and demographic parameters. We recognised that, although the Forest of Dean population is not fenced, it is sufficiently isolated so that immigration can be ignored. Furthermore, anecdotal evidence suggested that private hunting in the surrounding landscape appears to limit emigration.

## Results

### Parameterisation and evaluation

**Fig 2:**
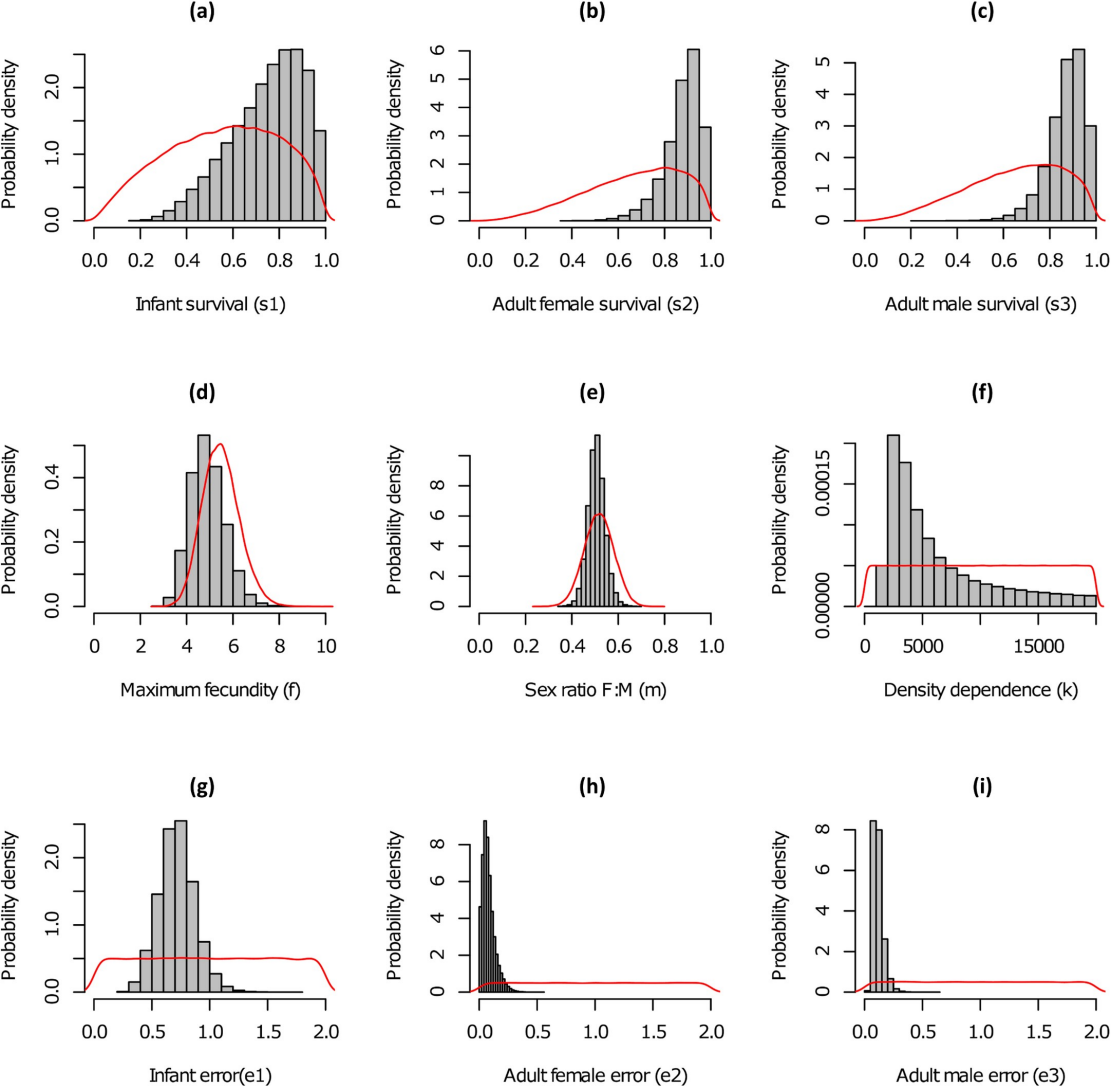
Parameter distributions. Posterior (bars) and prior (red lines) distributions for wild boar vital rate parameters.

**Fig 3:**
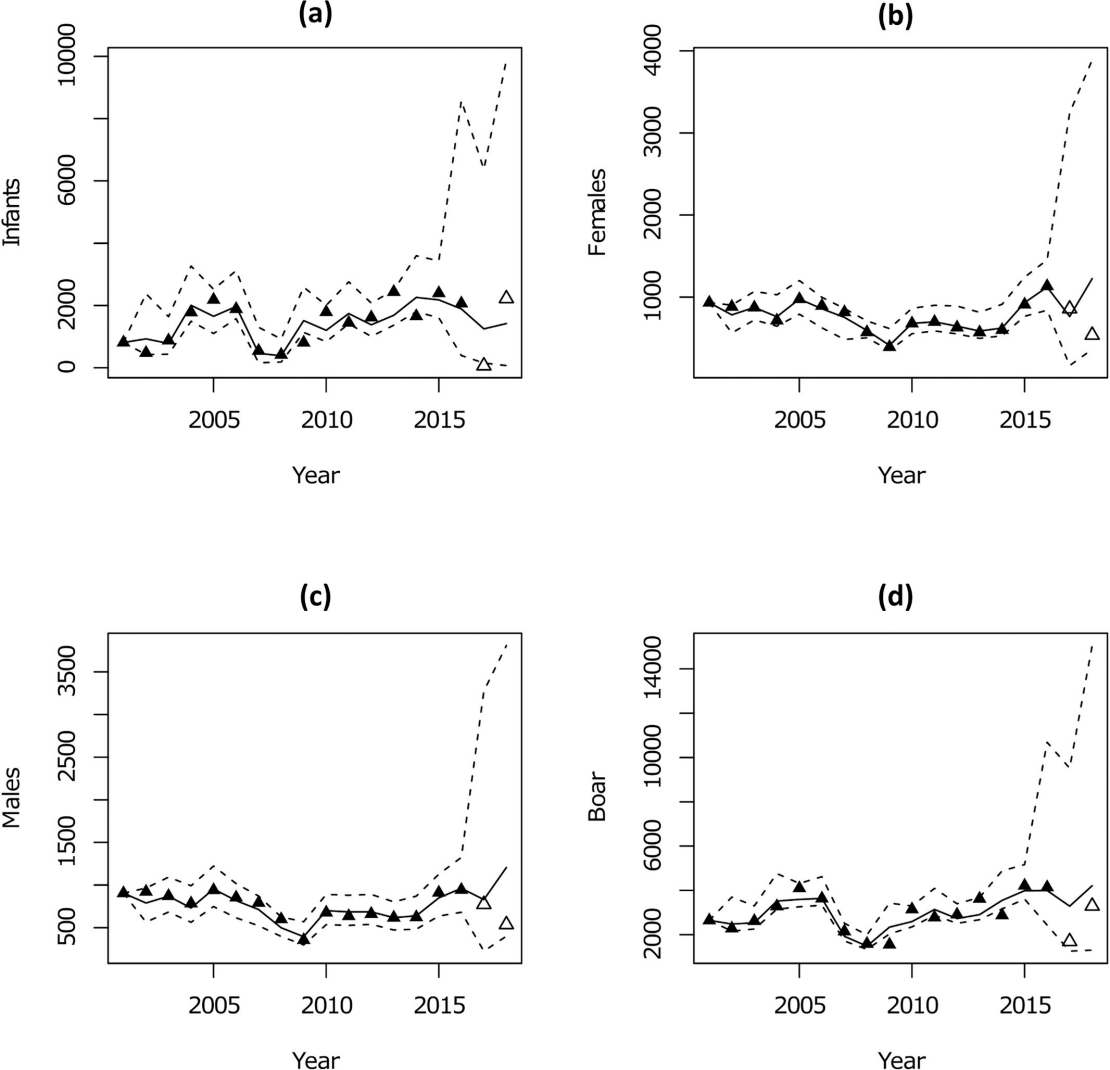
Model fit. Number of wild boar in different age and sex classes in Castelporziano predicted by the model versus number of animals estimated through distance sampling and demographic surveys at feeding stations. Filled and open triangles denote data included and excluded in model fitting respectively. Black lines show median population of boar against time. Dashed lines show 95% confidence interval.

We judged the MCMC to converge with the upper quantile of Gelman-Rubin diagnostic for all parameters less than 1.01. Posterior predictive checks showed no evidence of lack of fit with a *P*_*B*_ of 0.39. Comparison between prior and posterior parameter distributions (Fig 2) showed generally good agreement. Posterior survival probabilities were higher than respective priors with mean values of 0.75, 0.87 and 0.88 for juveniles, males and females (Table 2) and with an approximate increase of 0.2 compared to prior means. The posterior for maximum fecundity suggested marginally lower values compared to the prior with a mean of 4.9 and a reduction of 0.6 against the mean of the prior. For carrying capacity, estimates for Castelporziano suggested a posterior mean close to 6,000, equivalent to a density of approximately 100 boar/km^2^, at which reproduction and consequently growth rate are noticeably impacted by resource competition. With regard to the stochastic component of the model process, the variance was highest for juveniles with mean of 0.72, substantially greater than that estimated for males (0.12) or females (0.08). It is perhaps unsurprising therefore that compared to adults, which showed a close fit, juvenile populations appeared more difficult to predict (Fig 3). Overall, median predictions indicated the slow trend of population growth seen over the period 2001–2016 continuing with record numbers in 2018 following an initial dip in 2017. The wide intervals on long-term number of animals reflect the substantial uncertainty about the growth of the population in the future.

**Table 2:** Summary of parameter estimates.

Parameter	Mean	Median	SD	2.5% BCI	97.5% BCI
Carrying capacity (*k*)	6245	5000	4473	1814	18167
Fecundity (*f*)	4.90	4.85	0.74	3.61	6.50
Juvenile sex ratio (*m*)	0.50	0.50	0.04	0.43	0.58
Juvenile survival (*s*_*I*_)	0.75	0.77	0.16	0.39	0.97
Female survival (*s*_*F*_)	0.88	0.90	0.08	0.68	0.98
Male survival (*s*_*M*_)	0.87	0.89	0.08	0.68	0.98
Juvenile process variance (*σ*_*I*_)	0.72	0.71	0.16	0.42	1.05
Female process variance (*σ*_*F*_)	0.08	0.07	0.06	0.01	0.22
Male process variance (*σ*_*M*_)	0.12	0.11	0.04	0.06	0.22

Estimates of model parameters and 95% equal tailed Bayesian credible intervals (BCI).

### Simulation experiments

**Fig 4:**
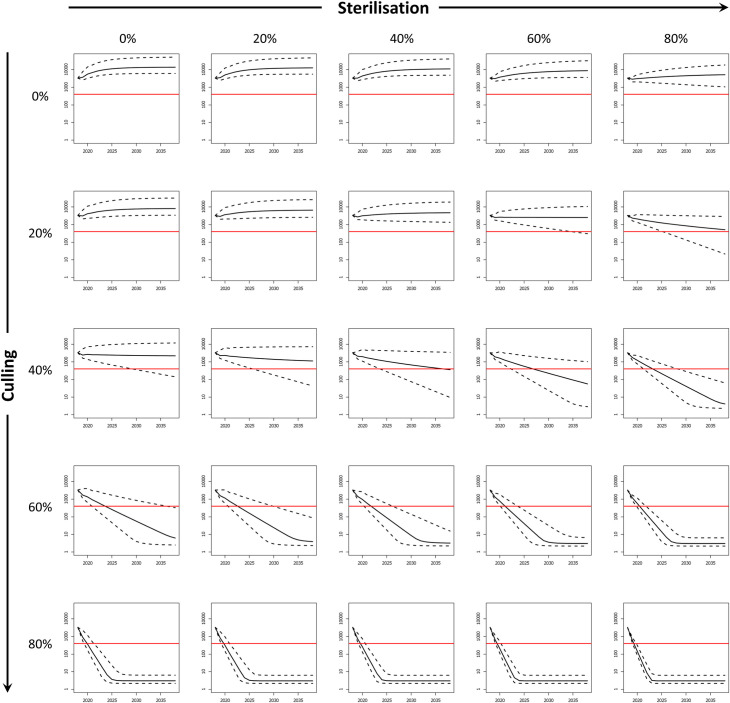
Simulation experiments for Castelporziano. Effects of various levels of culling and fertility control (0, 20, 40, 60 and 80%) on wild boar number (log-transformed) in Castelporziano. Black lines show median population of boar against time. Dashed lines show the 95% credible interval. Solid red line denotes nominal target population of 400 of wild boar.

In the absence of control the wild boar population in Castelporziano showed a maximum annual population growth rate of 1.51 (equivalent to an intrinsic growth rate of 0.41). Fertility control alone was not effective to reduce this population to 400 animals in 20 years (Fig 4). Reductions through culling alone were much more rapid although a culling rate at or below 20% failed to halt population growth. At 40% culling, the model suggested marginal population decline with a possibility, albeit small, probability of achieving the target reduction to 400 animals within 20 years. Culling above 60% showed rapid decline guaranteeing (97.5% BCI) reductions below the target threshold of 400 within the simulated period.

The addition of fertility control to complement culling increased the likelihood of reducing the population below 400 wild boar. Culling at 20% together with at least 60% contraception produced a decline in population but did not guarantee reducing the number of wild boar to the target 400 within 20 years, even with 80% of the animals rendered infertile. Crucially, the addition of at least 40% contraception to 40% culling resulted in at least a 50% likelihood of achieving the target reduction in 20 years, which would not be achieved by culling alone. The highest level (80%) of contraception was estimated to achieve the target reduction (97.5% BCI) within 10 years. For 60% culling, contraception reduced the time to target (97.5% BCI) from 18 years to 12, 8, 6 and 5 years for 20, 40, 60 and 80% contraception respectively. The addition of fertility control to 80% culling showed a negligible benefit.

**Fig 5:**
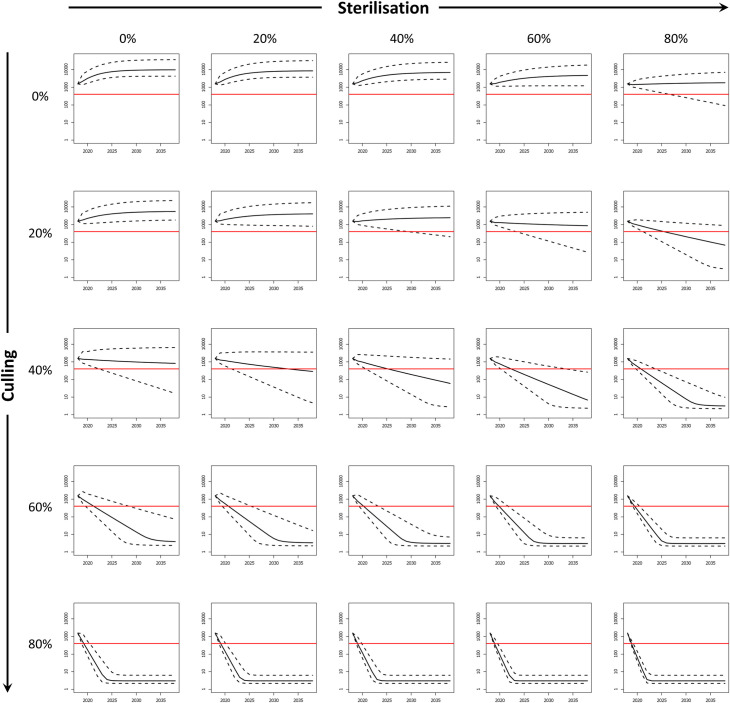
Simulation experiments for the Forest of Dean. Effects of various levels of culling and fertility control (0, 20, 40, 60 and 80%) on wild boar number (log-transformed) in the Forest of Dean, assuming added mortality due to road traffic accidents and to culling of animals that emigrate from the area. Black lines show median population of boar against time. Dashed lines show the 95% credible interval. Solid red line denotes nominal target population of 400 wild boar.

For the Forest of Dean (Fig 5), the growth rate in the absence of control was marginally smaller than Castelporziano at 1.44 (equivalent to an intrinsic 0.36), reflecting the additional mortality from RTAs but also the lower density (and therefore greater fecundity) of the population. Again, fertility control alone did not reliably produce population reduction, despite this greater mortality of adults. Only when applied at 80% was a population decline observed but not rapidly enough to reach the target population in all but a few repetitions (greater than 2.5% but less than 50%). However, combined with 40% culling, contraception levels of at least 60% guaranteed sufficiently rapid reduction to achieve the target population in 15 and 8 years with 60% and 80% of contraception respectively. As for Castelporziano, the addition of fertility control to culling at 60% did not markedly improve the median time to reach the target population but did improve the reliability with which this outcome was achieved. At this level of culling, a reduction to 400 wild boar was guaranteed (97.5% BCI) in 10 years (no contraception) and in 8, 5, 4 and 4 years assuming contraception levels of 20, 40, 60 and 80% respectively. This indicated that integrating 40% of fertility control with 60% culling would half the time to reach the target reduction in population size. The application of fertility control with culling of 80% did not provide any notable benefit.

## Discussion

This study is the first to use empirical data on both actual number of wild boar counted and on numbers removed from a closed population to model the impact of culling and fertility control on population size. The model, initially developed to examine the effects of culling on an isolated wild boar population (Preserve of Castelporziano) showed a close fit with the empirical data collected for 16 years on the number of adult male and female wild boar in this area. In particular, population trends predicted by the model were consistent with the number of wild boar estimated in the area with different methods (NLTS and CMR) under a culling regime that removed about 30% of the total population each year. This suggested that the outputs of the model, aimed at estimating the effects of different population control options on wild boar numbers, are plausible as they reflect real-world scenarios. The patterns predicted by the model for juveniles had comparatively larger confidence intervals than those for adult animals. This is likely due to significant year-to-year differences in the number of births in wild boar populations, as both litter size and proportion of females reproducing are very variable and depend on the availability of high-energy food such as acorns (e.g. [7, 75, 76]).

The novelty of the model consisted in simultaneously applying and comparing different levels of fertility control and culling to wild boar populations of known size and closed to immigration. The results showed that, whilst fertility control on its own was not sufficient to achieve the target reduction in wild boar number, adding fertility control to culling was more effective than culling alone. In particular, even in an area where natural mortality is relatively low, such as Castelporziano [40], the model predicted that 40% culling combined with 60% and 80% of sterilisation would achieve the target population of 400 wild boar in 15 and 8 years respectively, whilst 60% culling alone would take 16 years. If the level of culling was increased to 60%, the target number of wild boar would be achieved in 8, 5, and 4 and 4 years assuming 20, 40, 60 and 80% females were rendered infertile respectively.

The model was based on conservative estimates of population parameters, such as relatively high survival and maximum fecundity of wild boar. Data on fecundity included the numbers of embryos per female, although this is known to overestimate recruitment. For instance, Náhlik & Sandor [53] found that neonatal mortality in summer reduced litter size from 6.7 embryos per sow to approximately 3 juveniles per female observed a few months later. Similarly, Fruziński et al. [77] showed that litter size decreased through the year from 6.2 to 4.6 juveniles per sow between May and October respectively. At population sizes similar to those observed in Castelporziano the average fecundity predicted by the model is 3 juveniles per female. Accounting for juvenile survival this means average recruitment of approximately 2 juveniles per female.

Therefore it is likely that in most instances a reduction of wild boar numbers could be achieved sooner or with relatively lower levels of fertility control and/or culling than with those predicted by the model. Indeed, when the model was applied to the Forest of Dean, with an additional 10% of mortality rate due to road traffic accidents, contraception carried out in conjunction with 60% culling reduced the time to achieve 400 wild boar from 10 years (no contraception) to 8, 5, 4 and 4 years for levels of 20, 40, 60 and 80% of fertility control respectively. As for Castelporziano, fertility control alone did not produce population reduction in the Forest of Dean, despite the greater mortality of adults. However, even when combined with 40% culling, contraception guaranteed sufficiently rapid reduction to achieve the target population in 15 and 8 years for 60% and 80% contraception respectively.

The moderate levels of culling modelled in this study, namely 40%, is similar to the estimated 30% that in the last decades maintained a stable wild boar population in the Castelporziano Preserve, with densities of 22.6 ± 5.6 to 53.1 ± 8.1 per km^2^ (estimated with NLTS). Similar densities and levels of culling (21.1 per km^2^ and 25% respectively) were also recorded for the Forest of Dean. The model’s predictions on the effects of culling on wild boar numbers are consistent with previous studies suggesting that, in populations open to immigration, between 55 and 70% of a wild boar population should be removed each year to suppress population growth [8, 17, 35].

The results of our model broadly agree with the conclusions of a recent study that assessed the effects of incorporating hypothetical levels of fertility control with realistic culling intensities to reduce numbers of feral swine [39]. In simulated populations closed to immigration, this study found that annual culling of 20–60% led to reduction in number of animals (50–100% after 4 years) depending on the growth rate (considered annual growth rates between 1.3–2.43); our results (based on an equivalent growth rate of ~1.5) have suggested that population decline begins to occur with culling of 20–40% and is rapid above 60% with ~90% reductions possible within 4 years. The study [39] also showed that adding moderate levels of fertility control (40%) caused a substantially higher rate of population reduction; 50–70% more reduction over 4 years than culling alone. In agreement, our results showed that adding 40% fertility control to 60% culling halved the time to reach our population target and, when considered over an identical time period (4 years of management), could increase population reduction by as much as 43% (59% reduction with fertility control compared to 16% with culling alone based on the 97.5% BCI). Similar results were also obtained by a model on white-tailed deer (*Odocoileus virginianus*) [48], which concluded that treating annually 40% of the females with a contraceptive, once culling had reduced numbers to a desired target, was effective to maintain the population below carrying capacity. Our results also agree with a previous study in a Mediterranean urban area [35] which suggested that even high levels of fertility control (e.g. >70% of females made infertile), on its own, did not affect population size and trends.

Achieving the levels of fertility control highlighted in these studies will depend on the availability of and type of contraceptives, on the duration of induced infertility and on the feasibility of delivering these contraceptives to large numbers of animals. Whilst in our model fertility control may appear unfavourable compared to culling (i.e. 40% culling required versus 80% sterilisation) it may prove more cost effective, particularly if an oral contraceptive (currently not available) could be used and in contexts where hunters are unable or unwilling to reduce population size. Injectable contraceptives are already available that may render wild boar infertile for at least 4 to 6 years after a single injection [33]. These contraceptives, that obviously require capturing animals, might be considered in contexts, such as urban areas, where culling is infeasible or illegal or socially unacceptable.

Oral contraceptives are not yet available for wild boar. When these compounds will be formulated, they will have the potential to affect non-target species. Thus contraceptives administered in baits should be delivered through wild boar-specific bait dispensers. These devices, such as the Boar-Operated-System (BOS) already exist [78, 79] and could be employed to deliver oral contraceptives.

Our study was based on isolated populations of wild boar, closed to immigration by a fence (Castelporziano Preserve) or geographically isolated from others (Forest of Dean). The results are thus specific to these populations as other studies (e.g. [39, 48]) suggested that the effects of fertility control integrated with culling are less pronounced in open populations compared to closed ones. Whilst wild boar populations occur as a continuum across several countries and over vast areas, such as in mainland Europe, Eastern Australia and South-East USA [2, 3, 80], new isolated populations of illegally introduced animals continue to appear. The results of our model might be used to manage these isolated populations, particularly in areas where culling is not regarded feasible, cost-effective or acceptable. However, we recognise that where environmental conditions are substantially different to those of our study sites here (which we argue are sufficiently similar to be considered equivalent) more detailed understanding of how such factors impact the model parameterisation, particularly carrying capacity, is required. It is hoped that projects such as ENETWILD [81] which aims to estimate wild boar populations across Europe will provide the insights necessary to inform explicit site-specific description in the model, extending its direct transferability.

In conclusion, our model suggested that in isolated populations of wild boar adding fertility control to culling will accelerate population decline, in agreement with previous theoretical studies on feral swine [39]. The data used for our model are unique as we could validate the model against known population size and rates of animal removal, thus increasing our confidence in the model’s predictions. Our work emphasised the importance of collecting these data to optimise allocation of resources by local managers and to inform adaptive management of populations of wild boar. As wild boar and feral swine numbers grow, effective and sustainable approaches will become a priority to decrease densities and impacts on human activities and biodiversity [82, 83], particularly as hunting pressure, which is the main source of mortality in wild boar, decreases [6]. For the majority of hunters, culling is a recreational activity, and for game keepers and hunting organisations wild boar is an economically important resource that is purposely managed, protected and exploited, often with remarkable investment of money, time and labour [84]. Employing “community empowered” methods driven by volunteers and based on culling will have costs that should be quantified accurately, especially when “population reduction” is an objective not fully shared by all stakeholders. The actual success of programmes aimed at reducing the impact of wild boar and feral swine should be measured by quantifying the extent of decreases of the environmental and/or economic impacts achieved with the control methods adopted. Future studies should focus on context-specific feasibility, costs, public acceptance and impact of integrating culling with fertility control to manage populations of wild boar and feral swine. Moreover, future research should investigate the effects of culling and fertility control on spatial and social behaviour of wild boar and feral swine as it is likely that the two methods could have very different effects on animals. For instance, fertility control might result in the “placeholder effect”, with infertile animals occupying areas unavailable to fertile immigrants and with a relatively small social disruption compared to culling [32, 39]. This might have important consequences for dispersal and for contact rate between individuals, particularly relevant in the context of disease outbreaks.

## Supporting information

S1 File File containing the population, demographic and removal data used to inform the model fit. (XLSX)

S2 File Archive containing all R scripts and resources required to run the model. (ZIP)

S1 Fig Plots for each model parameter showing trace (convergence) and density (posterior distribution). (PDF)

## References

[1] Ballari SA, Cuevas MF, Cirignoli S, Valenzuela AE. Invasive wild boar in Argentina: using protected areas as a research platform to determine distribution, impacts and management. Biological Invasions, 2015 17(6): p. 1595–1602.

[2] Sjarmidi A, Gerard JF. Autour de la systématique et la distribution des suidés. Monitore Zoologico Italiano—Italian Journal of Zoology. 1988; 22(4):415–448.

[3] Snow NP, Jarzyna MA, VerCauteren KC. Interpreting and predicting the spread of invasive wild pigs. Journal of Applied Ecology. 2017; 54(6):2022–2032.

[4] Bevins SN, Pedersen K, Lutman MW, Gidlewski T, Deliberto TJ. Consequences associated with the recent range expansion of nonnative feral swine. BioScience. 2014; 64(4):291–299.

[5] Frauendorf M, Gethöffer F, Siebert U, Keuling O. The influence of environmental and physiological factors on the litter size of wild boar (Sus scrofa) in an agriculture dominated area in Germany. Science of The Total Environment. 2016; 541:877–882. 10.1016/j.scitotenv.2015.09.128 26437356

[6] Massei G, Kindberg J, Licoppe A, Gačić D, Šprem N, Kamler J, et al, Wild boar populations up, numbers of hunters down? A review of trends and implications for Europe. Pest Management Science. 2015; 71(4):492–500. 10.1002/ps.3965 25512181

[7] Bieber C, Ruf T. Population dynamics in wild boar *Sus scrofa*: ecology, elasticity of growth rate and implications for the management of pulsed resource consumers. Journal of Applied Ecology. 2005; 42(6):1203–1213.

[8] Keuling O, Baubet E, Duscher A, Ebert C, Fischer C, Monaco A, et al, Mortality rates of wild boar *Sus scrofa L*. in central Europe. European Journal of Wildlife Research. 2013; 59(6):805–814.

[9] Anderson A, Slootmaker C, Harper E, Holderieath J, Shwiff SA. Economic estimates of feral swine damage and control in 11 US states. Crop Protection. 2016; 89:89–94.

[10] Barrios-Garcia MN, Ballari SA. Impact of wild boar (*Sus scrofa*) in its introduced and native range: a review. Biological Invasions. 2012; 14(11):2283–2300.

[11] Bueno CG, Barrio IC, García-González R, Alados CL, Gómez-García D. Does wild boar rooting affect livestock grazing areas in alpine grasslands?. European Journal of Wildlife Research. 2010; 56(5):765–770.

[12] Hone J. Feral pigs in Namadgi National Park, Australia: dynamics, impacts and management. Biological Conservation. 2002; 105(2):231–242.

[13] Massei G, Genov PV. The environmental impact of wild boar. Galemys. 2004; 16(1):135–145.

[14] Welander J. Spatial and temporal dynamics of wild boar (*Sus scrofa*) rooting in a mosaic landscape. Journal of Zoology. 2000; 252(2):263–271.

[15] Cahill S, Llimona F, Cabañeros L, Calomardo F. Characteristics of wild boar (*Sus scrofa*) habituation to urban areas in the Collserola Natural Park (Barcelona) and comparison with other locations. Animal Biodiversity and Conservation. 2012; 35(2):221–233.

[16] Stillfried M, Fickel J, Börner K, Wittstatt U, Heddergott M, Ortmann S, et al Do cities represent sources, sinks or isolated islands for urban wild boar population structure? Journal of Applied Ecology. 2017; 54(1):272–281.

[17] Bengsen AJ, Gentle MN, Mitchell JL, Pearson HE, Saunders GR. Impacts and management of wild pigs *Sus scrofa* in Australia. Mammal Review. 2014; 44(2):135–47.

[18] Guo X, Claassen GD, Oude Lansink AG, Loeffen W, Saatkamp HW. Economic analysis of Classical Swine Fever surveillance in the Netherlands. Transboundary and Emerging Diseases. 2016; 63(3):296–313. 10.1111/tbed.12274 25213149

[19] Halasa T, Bøtner A, Mortensen S, Christensen H, Toft N, Boklund A. Simulating the epidemiological and economic effects of an African swine fever epidemic in industrialized swine populations. Veterinary Microbiology. 2016; 193:7–16. 10.1016/j.vetmic.2016.08.004 27599924

[20] Massei G, Roy S, Bunting R. Too many hogs? A review of methods to mitigate impact by wild boar and feral hogs. Human-Wildlife Interactions. 2011; 5(1):79–99.

[21] Parkes JP, Ramsey DS, Macdonald N, Walker K, McKnight S, Cohen BS, et al Rapid eradication of feral pigs (*Sus scrofa*) from Santa Cruz Island, California. Biological Conservation. 2010; 143(3):634–641.

[22] Sharp T, Saunders G. Model code of practice for the humane control of feral pigs. NSW Department of Primary Industries, Orange, Australia. 2004.

[23] Cremasco P, Gentle M, Wilson C, Di Bella L, Buckman M. Feral pig baiting with fruit in the wet tropics. In 5th Queensland Pest Animal Symposium 2016; 103–106.

[24] Bengsen AJ, Sparkes J. Can recreational hunting contribute to pest mammal control on public land in Australia? Mammal Review. 2016; 46(4):297–310.

[25] Keuling O, Strauß E, Siebert U. Regulating wild boar populations is “somebody else's problem”!—Human dimension in wild boar management. Science of The Total Environment. 2016; **554–**555:311–319. 10.1016/j.scitotenv.2016.02.159 26956178

[26] Centner TJ, Shuman RM. Governmental provisions to manage and eradicate feral swine in areas of the United States. AMBIO. 2015; 44(2):121–130. 10.1007/s13280-014-0532-9 24845195 PMC4329133

[27] Giacomelli S, Gibbert M, Viganò R. Community empowerment for managing wild boar: a longitudinal case study of northern Italy 2001–2018. Ecology and Society. 2018; 23(4):12.

[28] Hone J. Applied population and community ecology: the case of feral pigs in Australia. John Wiley & Sons 2012.

[29] Twigg LE, Lowe T, Everett M, Martin G. Feral pigs in north-western Australia: population recovery after 1080 baiting and further control. Wildlife Research. 2006; 33(5):417–425.

[30] Shapiro L, Eason C, Bunt C, Hix S, Aylett P, MacMorran D. Efficacy of encapsulated sodium nitrite as a new tool for feral pig management. Journal of Pest Science. 2016; 89(2):489–495.

[31] Conejero C, Castillo-Contreras R, González-Crespo C, Serrano E, Mentaberre G, Lavín S, et al Past experiences drive citizen perception of wild boar in urban areas. Mammalian Biology. 2019; 96(1):68–72.

[32] Massei G, Cowan D. Fertility control to mitigate human-wildlife conflicts: a review. Wildlife Research. 2014; 41(1):1–21.

[33] Massei G, Cowan DP, Coats J, Bellamy F, Quy R, Pietravalle S, et al Long-term effects of immunocontraception on wild boar fertility, physiology and behaviour. Wildlife Research. 2012; 39(5):378–385.

[34] Oliviero C, Lindh L, Peltoniemi O. Immunocontraception as a possible tool to reduce feral pig populations: recent and future perspectives. Journal of Animal Science. 2019; 97(6):2283–2290. 10.1093/jas/skz066 30753509 PMC6541807

[35] Gonzalez-Crespo C, Serrano E, Cahill S, Castillo-Contreras R, Cabañeros L, López-Martín JM, et al Stochastic assessment of management strategies for a Mediterranean peri-urban wild boar population. PLoS ONE. 2018; 13(8):e0202289 10.1371/journal.pone.0202289 30157225 PMC6114779

[36] Lange M. Alternative control strategies against ASF in wild boar populations. EFSA Supporting Publications 2015; 12(7):843E.

[37] Focardi S, Toso S, Pecchioli E. The population modelling of fallow deer and wild boar in a Mediterranean ecosystem. Forest Ecology and Management. 1996; 88(1):7–14.

[38] Imperio S, Focardi S, Santini G, Provenzale A. Population dynamics in a guild of four Mediterranean ungulates: density‐dependence, environmental effects and inter‐specific interactions. Oikos. 2012; 121(10):1613–1626.

[39] Pepin KM, Davis AJ, Cunningham FL, VerCauteren KC, Eckery DC. Potential effects of incorporating fertility control into typical culling regimes in wild pig populations. PLoS ONE. 2017; 12(8):e0183441 10.1371/journal.pone.0183441 28837610 PMC5570275

[40] Focardi S, Gaillard JM, Ronchi F, Rossi S. Survival of wild boars in a variable environment: unexpected life-history variation in an unusual ungulate. Journal of Mammalogy. 2008; 89(5):1113–1123.

[41] Wilson CJ. The establishment and distribution of feral wild boar (*Sus scrofa*) in England. Wildlife Biology in Practice. 2014; 10(3):1–6.

[42] Franzetti B, Ronchi F, Marini F, Scacco M, Calmanti R, Calabrese A, et al Nocturnal line transect sampling of wild boar (*Sus scrofa*) in a Mediterranean forest: long-term comparison with capture–mark–resight population estimates. European Journal of Wildlife Research. 2012; 58(2):385–402.

[43] Gamelon M, Focardi S, Baubet E, Brandt S, Franzetti B, Ronchi F, et al Reproductive allocation in pulsed-resource environments: a comparative study in two populations of wild boar. Oecologia. 2017; 183(4):1065–1076. 10.1007/s00442-017-3821-8 28154966

[44] Buckland ST, Anderson DR, Burnham KP, Laake JL, Borchers DL, Thomas L. Advanced distance sampling (Vol. 2). Oxford University Press, Oxford, UK 2004.

[45] Gill RMA, Thomas ML, Stocker D. The use of portable thermal imaging for estimating deer population density in forest habitats. Journal of Applied Ecology. 1997; 34(5):1273–1286.

[46] Smart JCR, Ward AI, White PCL. Monitoring woodland deer populations in the UK: an imprecise science. Mammal Review. 2004; 34(1‐2):99–114.

[47] Gill RMA, Waeber K. Feral wild boar and deer in the Forest of Dean. Population surveys in the public Forest Estate 2018. Forest Research, UK 2018.

[48] Raiho AM, Hooten MB, Bates S, Hobbs NT. Forecasting the effects of fertility control on overabundant ungulates: white-tailed deer in the National Capital Region. PLoS ONE. 2015; 10(12):e0143122 10.1371/journal.pone.0143122 26650739 PMC4674220

[49] Leslie PH. On the use of matrices in certain population mathematics. Biometrika. 1945; 33(3):183–212. 21006835 10.1093/biomet/33.3.183

[50] Holland EP, Burrow JF, Dytham C, Aegerter JN. Modelling with uncertainty: Introducing a probabilistic framework to predict animal population dynamics. Ecological Modelling. 2009; 220(9–10):1203–1217.

[51] Venables WN, Ripley BD. Modern Applied Statistics with S. Fourth Edition Springer, New York, USA 2002.

[52] R Core Team. R: A language and environment for statistical computing. R Foundation for Statistical Computing, Vienna, Austria https://www.R-project.org/. 2017.

[53] Náhlik A, Sándor G. Birth rate and offspring survival in a free-ranging wild boar *Sus scrofa* population. Wildlife Biology. 2003; 9(4):37–42.

[54] Ahmad E, Brooks JE, Hussain I, Khan MH. Reproduction in Eurasian wild boar in central Punjab, Pakistan. Acta Theriologica. 1995; 40:163–163.

[55] Boitani L, Trapanese P, Mattei L. Demographic patterns of a wild boar (*Sus scrofa* L.) population in Tuscany, Italy. Journal of Mountain Ecology. 1995; 3:197–201.

[56] Briedermann L. Schwarzwild, Second Edition. VEB Deutscher Landwirtschaftsverlag, Berlin, Germany. 1990.

[57] Gill RMA, Ferryman M. Feral wild boar and deer in the Forest of Dean. Survey and population projections in the public Forest Estate 2015. Forest Research, UK 2015.

[58] Martys M. Gehegebeobachtungen zur Geburts- und Reproduktionsbiologie des Europäischen Wildschweins (*Sus scrofa* L.). Zeitschrift für Säugetierkunde. 1982; 47:100–113.

[59] Moretti M. Birth distribution, structure and dynamics of a hunted mountain population of wild boars (*Sus scrofa L*.), Ticino, Switzerland. Journal of Mountain Ecology. 1995; 3:192–196.

[60] Oloff HB. Zur Biologie and Ökologie des Schwarzwildes. Dr Paul Schöps-Verlag, Leipzig, Germany 1951.

[61] Servanty S, Gaillard JM, Allainé D, Brandt S, Baubet E. Litter size and fetal sex ratio adjustment in a highly polytocous species: the wild boar. Behavioral Ecology. 2007; 18(2):427–432.

[62] Fernández-Llario P, Carranza J, Mateos-Quesada P. Sex allocation in a polygynous mammal with large litters: the wild boar. Animal Behaviour. 1999; 58(5):1079–1084. 10.1006/anbe.1999.1234 10564610

[63] Neet CR. Population dynamics and management of *Sus scrofa* in western Switzerland: a statistical modelling approach. Journal of Mountain Ecology. 1995; 3:188–191.

[64] Gamelon M, Focardi S, Gaillard JM, Gimenez O, Bonenfant C, Franzetti B, et al Do age‐specific survival patterns of wild boar fit current evolutionary theories of senescence?. Evolution. 2014; 68(12):3636–3643. 10.1111/evo.12519 25180915

[65] Jezierski W. Longevity and mortality rate in a population of wild boar. Acta Theriologica. 1977; 22(24):337–348.

[66] Lange M, Guberti V, Thulke H. Understanding ASF spread and emergency control concepts in wild boar populations using individual‐based modelling and spatio‐temporal surveillance data. EFSA Supporting Publications. 2018; 15(11):1521E.

[67] Servanty S, Gaillard JM, Ronchi F, Focardi S, Baubet E, Gimenez O. Influence of harvesting pressure on demographic tactics: implications for wildlife management. Journal of Applied Ecology. 2011; 48(4):835–843.

[68] Toïgo C, Servanty S, GAILLARD JM, Brandt S, Baubet E. Disentangling natural from hunting mortality in an intensively hunted wild boar population. The Journal of Wildlife Management. 2008; 72(7):1532–1539.

[69] Plummer M. JAGS: A program for analysis of Bayesian graphical models using Gibbs sampling. In proceedings of The 3rd International Workshop on Distributed Statistical Computing. 2003; 10.

[70] Plummer M. rjags: Bayesian Graphical Models using MCMC. R package version 4–6. http://CRAN.R-project.org/package = rjags. 2016.

[71] Brooks SP, Gelman A. General methods for monitoring convergence of iterative simulations. Journal of Computational and Graphical Statistics. 1998; 7(4):434–455.

[72] Gelman A, Rubin DB. Inference from iterative simulation using multiple sequences. Statistical Science. 1992; 7(4):457–472.

[73] Gelman A, Carlin JB, Stern HS, Dunson DB, Vehtari A, Rubin DB. Bayesian data analysis. Taylor & Francis, Florida, USA 2013.

[74] Moore N. The ecology and management of wild boar in southern England. Defra Report (VC0325). Department for Environment, Food and Rural Affairs, HM Government, UK 2004.

[75] Massei G, Genov PV, Staines BW. Diet, food availability and reproduction of wild boar in a Mediterranean coastal area. Acta Theriologica. 1996; 41:307–320.

[76] Mauget R, Campan R, Spitz F, Dardaillon M, Janeau G, Pepin D. Synthèse des connaissances actuelles sur la biologie du sanglier, perspectives de recherche. In Symposium International sur le Sanglier’.(Eds F. Spitz and D. Pepin.) 1984; 15–50.

[77] Fruziński B, Łabudzki L. Management of wild boar in Poland. Zeitschrift für Jagdwissenschaft. 2002; 48(1):201–207.

[78] Campbell TA, Long DB, Massei G. Efficacy of the Boar-Operated-System to deliver baits to feral swine. Preventive Veterinary Medicine. 2011; 98(4):243–249. 10.1016/j.prevetmed.2010.11.018 21176854

[79] Massei G, Coats J, Quy R, Storer K, Cowan DP. The boar‐operated‐system: a novel method to deliver baits to wild pigs. The Journal of Wildlife Management. 2010; 74(2):333–336.

[80] Bengsen AJ, West P, Krull CR. Feral pigs in Australia and New Zealand: range, trend, management and impacts of an invasive species, in Ecology, Evolution and Management of Wild Pigs and Peccaries. Implications for Conservation. Cambridge University Press, Cambridge, UK 2016.

[81] ENETWILD consortium, Acevedo P, Croft S, Fernandez-Lopez J, Scandura M, Apollonio M, et al Update of occurrence and hunting yield-based data models for wild boar at European scale: new approach to handle the bioregion effect. EFSA Supporting Publications 2020; 17(5):28.

[82] Graitson E, Barbraud C, Bonnet X. Catastrophic impact of wild boars: insufficient hunting pressure pushes snakes to the brink. Animal Conservation. 2019; 22(2):165–176.

[83] Oja R, Soe E, Valdmann H, Saarma U. Non-invasive genetics outperforms morphological methods in faecal dietary analysis, revealing wild boar as a considerable conservation concern for ground-nesting birds. PLoS ONE. 2017; 12(6):e0179463 10.1371/journal.pone.0179463 28594953 PMC5464655

[84] Guberti V, Khomenko S, Masiulis M, Kerba S. Handbook on African Swine Fever in wild boar and biosecurity during hunting. Handbook on African Swine Fever in wild boar and biosecurity during hunting. Standing Group of Experts on African swine fever in Europe under the GF-TADs umbrella. 2018.

