# Supplementary material for: Too many wild boar? Modelling fertility control and culling to reduce wild boar numbers in isolated populations
Source: PLoS One. 2020 Sep 18;15(9):e0238429. doi: 10.1371/journal.pone.0238429 (PMC7500663; doi:10.1371/journal.pone.0238429)
Supplement: S1 Fig — (PDF) [file pone.0238429.s003.pdf]

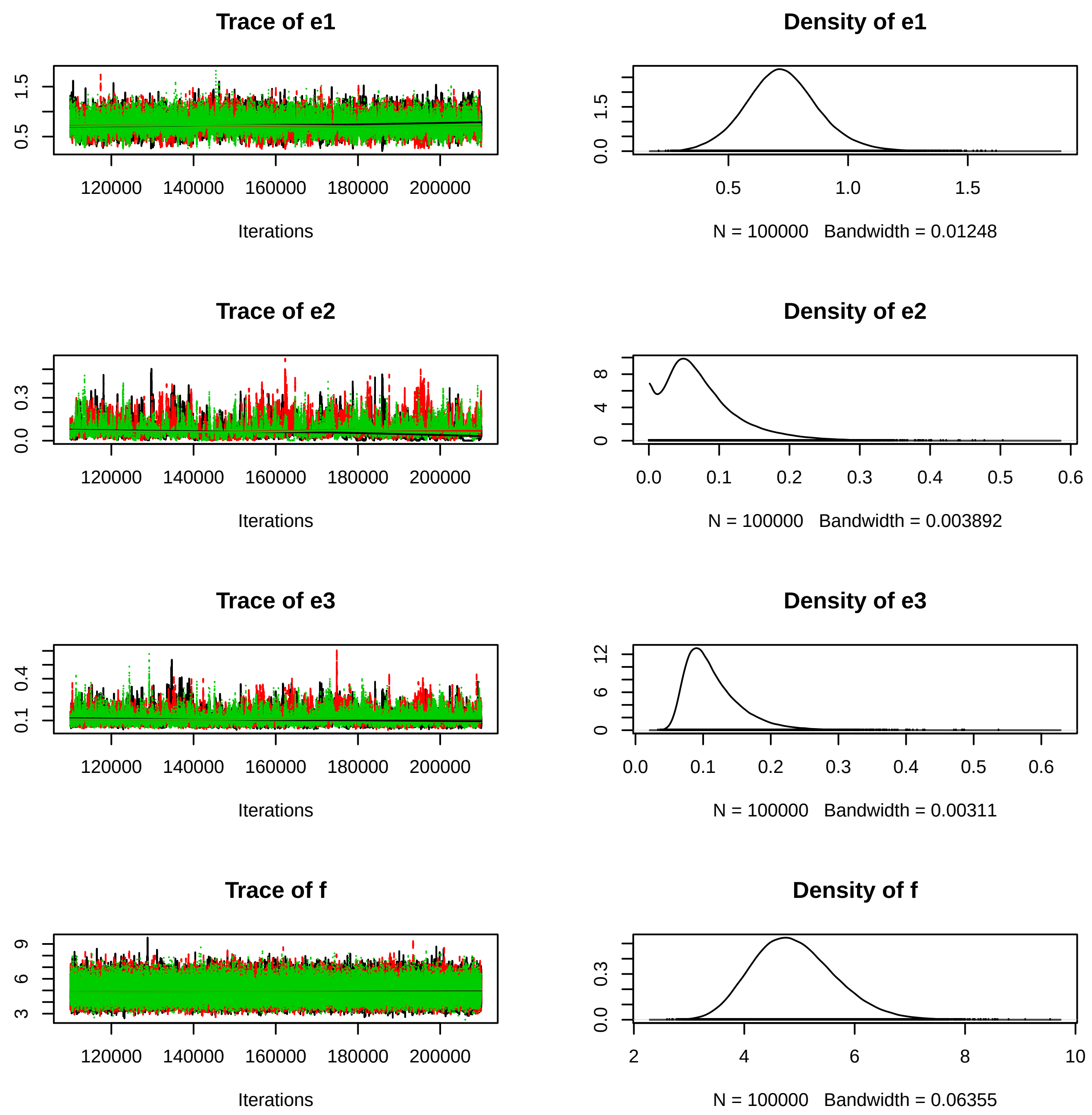

Trace of e1
Iterations
Density of e1
N = 100000 Bandwidth = 0.01248
Trace of e2
Iterations
Density of e2
N = 100000 Bandwidth = 0.003892
Trace of e3
Iterations
Density of e3
N = 100000 Bandwidth = 0.00311
Trace of f
Iterations
Density of f
N = 100000 Bandwidth = 0.06355

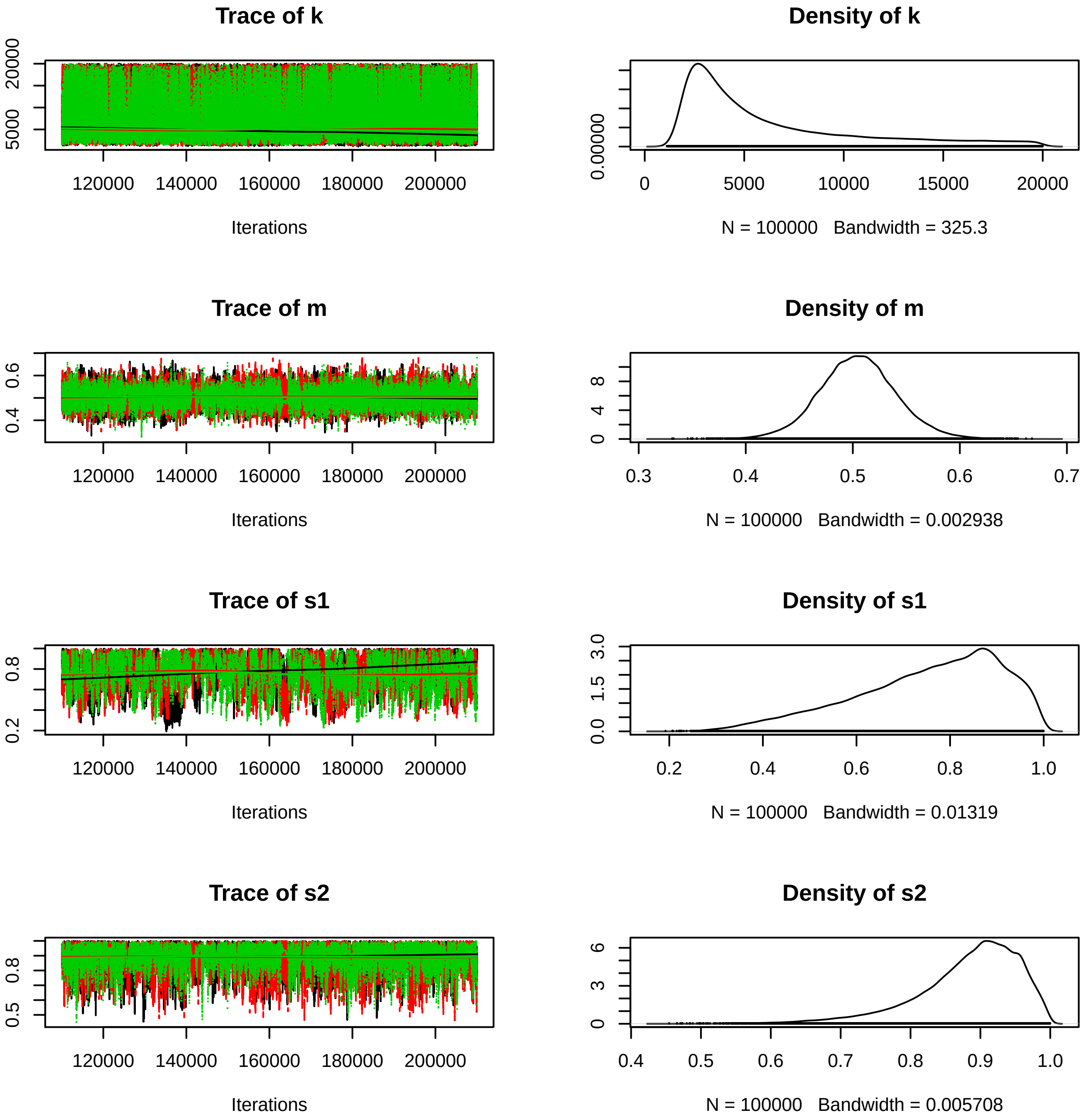

Trace of k
Iterations
Density of k
N = 100000 Bandwidth = 325.3
Trace of m
Iterations
Density of m
N = 100000 Bandwidth = 0.002938
Trace of s1
Iterations
Density of s1
N = 100000 Bandwidth = 0.01319
Trace of s2
Iterations
Density of s2
N = 100000 Bandwidth = 0.005708

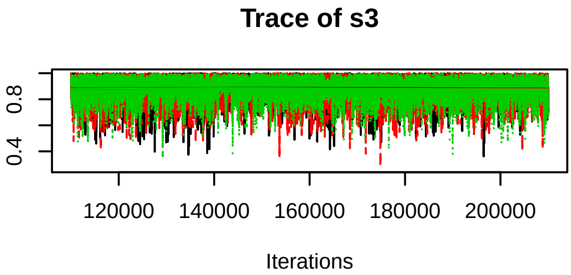

Trace of s3
0.4
0.8
120000
140000
160000
180000
200000
Iterations

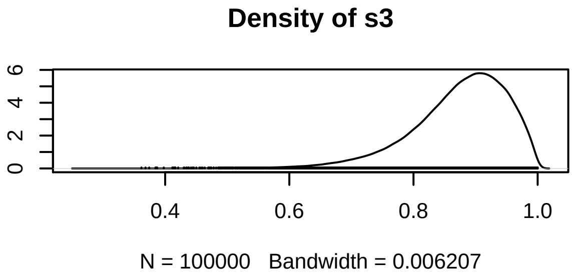

Density of s3
0
2
4
6
0.4
0.6
0.8
1.0
N = 100000 Bandwidth = 0.006207
